# Protect the Player, Protect the Game: Reflections from Ex-Professional Rugby Union Players on Law Changes, Protective Equipment, and Duty of Care in the Professional Game

**DOI:** 10.3390/jfmk7040091

**Published:** 2022-10-20

**Authors:** Ed Daly, Alexander D. Blackett, Alan J. Pearce, Lisa Ryan

**Affiliations:** 1 School of Science & Computing, Atlantic Technological University, H91 T8NW Galway, Ireland; 2 School of Life Sciences and Education, Staffordshire University, Stoke-on-Trent ST4 2DF, UK; 3 College of Sport, Health and Engineering, La Trobe University, Melbourne, VIC 3086, Australia

**Keywords:** concussion risk, duty of care, coaches, rugby union, professional rugby players

## Abstract

The emphasis of this study was to interview ex-professional male rugby union players (*n* = 23, mean age 35.5 ± 4.7 years) and discuss concussion management during their careers. In this study, two major themes were identified: (1) the duty of care to professional rugby union players by medical personnel, coaching staff, and owners of professional clubs and (2) the use of protective equipment and law changes to enhance player safety. In total, twenty-three ex-professional rugby union players were interviewed, and the majority (61%) had represented their countries at international test-level rugby. These interviews highlighted the belief that medical teams should be objective, independent entities within a professional rugby club. Furthermore, medical teams should not be in a position of being pressurised by head coaches, members of the coaching team, or club owners regarding return-to-play (RTP) protocols specific to concussion. The interviewees believed that they were pressured by coaches or members of the coaching team to play with concussion or concussive symptoms and other physical injuries. The results indicated that they had manipulated concussion testing themselves or with assistance to pass standard concussion testing protocols. The interviewees indicated that club owners have a duty of care to players even in retirement due to the high incidence of physical and mental injuries endured as a professional rugby player. Most participants indicated that a reduction in match playing time and reducing the amount of time engaged in contact training (workload volume) may assist in reducing concussion incidence. The participants suggested that changes to the current laws of the game or the use of protective equipment did not mitigate against concussion risk in the game of rugby union. The main limitation to the study is that participants had retired in the past ten years, and conditions for players may have changed. This study has highlighted that additional efforts are required by professional clubs to ensure the highest duty of care is delivered to current players and recently retired players.

## 1. Introduction

Rugby union transitioned to a professional sport in 1995, and this evolution meant that professional clubs could be formed from existing amateur clubs, and players could receive a salary [1]. The change to professionalism was turbulent, as many observers considered this a move away from traditional rugby values, such as participation being of a higher importance than winning [2]. In effect, these early days of professionalism led to well-publicised disputes between the owners of professional clubs and national governing bodies about access to players [3]. The cause of these disputes was primarily financial in nature. For example, during the 2016/17 season, the Rugby Football Union had to pay English Premiership Rugby EUR 244 million for using players to represent their countries at the international level [4].

Essentially, the level of finance that currently exists in rugby union via private investment and highly lucrative broadcasting deals means that the game has fundamentally changed since the advent of professionalism [4]. These pressures to balance financial performance off the field and sporting performance on the field remains a constant challenge. This is particularly evident for professional rugby union clubs, as the potential to generate revenue is not as high when compared to other professional sports such as association football (soccer) [5]. This results in a combination of circumstances that creates latent burdens on club owners, coaching staff, medical teams, and more importantly, the players who undergo the challenge of performing on a field of play on a weekly basis [6]. 

As rugby players are at the forefront of meeting these challenges, they are now exposed to multiple demands from a wide range of areas. Players are full-time professional athletes and are required to be in peak physical condition to meet the rigors of the game [7]. Since the beginning of professionalism, the anthropometry of players has evolved exponentially over time due to rigorous strength-training practices [8,9]. In addition to being physically ready, the stresses of training for competition and competing in matches should focus on individual workload monitoring for player welfare and adequate recovery time [10]. These collective requirements exact considerable physical and mental demands from players in terms of the workload volume, on-field performance, and exposure to injury risk [11]. 

Research by West et al. [12] found that injury severity increased in professional rugby union between 2002–2019, with tackling accounting for 43% of the overall injury burden (n = 10,851 injuries occurred). This research found that the incidence, severity, and burden of concussion increased from 2009–2010 onwards, and from 2011–2019, concussion was the most common injury recorded [12]. Many players (amateur and professional) choose to wear protective equipment to mitigate against impact and injury [6]. A recent study by Stokes et al. (2021) reported that the use of protective equipment had no significant impact on concussion incidence in professional rugby (adjusted odds ratio = 1.05, 95% CI: 0.71–1.56).

Athletes have been reported in the literature to not disclose concussions for several reasons. Pressure from fellow players, fear of being omitted from squads, being perceived as weak, or the precarious nature of playing contracts have all been reported as reasons for a lack of disclosure by players [13,14]. As professional rugby players may not disclose their concussions, it is incumbent on medical teams and coaching staff to identify abnormalities concerning the players’ health during training and competitive matches [15]. Exposure to further concussion injury risk or inaccurate on-field assessments is known to endanger the player to further injury [16]. There have been open discussions about concerns of medical teams, coaching staff, club owners, and player-representative bodies regarding the long-term health of current and retired players [17]. These concerns have been justified based on recent revelations about repetitive head impacts and long-term cognitive and neurological damage to ex-professional players [18,19]. The purpose of this current research was to gain the players’ perspectives on concussion management practices, including the collective duty of care from head coaches, coaching staff, medical teams, and owners of professional rugby club franchises.

## 2. Methods

### 2.1. Study Design

A reflexive thematic analysis approach was adopted for this research study using the Braun and Clarke methodology [20,21]. A semi-structured interview design was utilised to individually interview the ex-professional male rugby union players (*n* = 23) to ascertain their personal opinions regarding the duty of care that head coaches, coaching staff, medical teams, and club owners had towards professional players. All participants in this study had been employed as professional players and had retired from the game of rugby union within the last 10 years. The format for the interview questions were designed to establish their career as professional rugby players, including how their careers commenced, progressed, and ended. Questions on the frequency of career exposure to injury were incorporated along with specific questions related to concussions and physical injury. This in turn would provide their opinions on who was responsible for the duty of care with respect to concussion management.

### 2.2. Ethics and Procedure

Ethical approval was sanctioned for this study via the Research Sub-Committee of Academic Council of Atlantic Technological University (ATURSC_AC_23062020). The initial number of participants were identified via the lead researcher (ED) by distributing an open request alongside a detailed participant information sheet regarding the purpose of the study. The initial informal discussions were an occasion for the participants to discuss the objectives of the study and how the resulting data would be managed in a strictly confidential manner. Verbal consent was recorded for each participant before their individual interview occurred. As the interviews were semi-structured, the data that were collected from interviews that ranged from 25–70 min in duration. A standardized series of questions and supporting questions were applied across all the interviews. All information related to the study were fully anonymised to protect the ex-players from identification.

### 2.3. Participants Characteristics

The twenty-three male participants who had retired from playing professional rugby union at the time the interviews were facilitated. At the time of data collection, the ages of the players that were interviewed ranged from 29 years old to 43 years old (average age 35.5 ± 4.7 years). Participants were interviewed from a wide distribution of countries including both the Northern and Southern Hemispheres to obtain broad perspective on this topic. The following countries were represented: Ireland (*n* = 17), England (*n* = 1), Scotland (*n* = 3), and Australia (*n* = 2). From this group of players (*n* = 23), 14 had represented their national team at full-test-level rugby union (61%). The mean career duration in professional rugby for the cohort was 9.3 ± 2.7 years, and the average age at the time of retirement from the game was 30.8 ± 2.9 years (≤10 years since retirement). With respect to the positions that they played during their careers (i.e., “forwards” and “backs”), 70% were forwards, and 30% were backs. Within these two broad playing divisions, the forwards had a breakdown as follows: front row (*n* = 6), second row (*n* = 2), and back row (*n* = 8). The backs had the following breakdown: winger (*n* = 2), centres (*n* = 3), and scrum half (*n* = 2).

### 2.4. Sampling and Eligibility Criteria

For the purposes of this study, an exponential non-discriminative snowball sampling method was utilised, whereby the initial participants who volunteered provided multiple referrals [22]. Each ex-professional player that volunteered through the referral process was investigated until primary data from sufficient samples were collected, and information redundancy was reached [23]. Participants were clearly informed that they had no obligation to introduce any other potential participants. The interviews recorded a standardised sequence of responses due to the semi-structured nature of the process across the participants. This permitted the research team to identify common themes and responses from the cohort of participants.

### 2.5. Data Collection

The replies to the interview questions were used to gather evidence of their personal experiences. These experiences described their position and opinions on how club medical teams, head coaches, and owners of professional rugby clubs managed concussion. These personal incidents occurred during their professional careers and included the observed experiences of fellow professional players that they witnessed. This study sought to add the “player’s perspective” to the information that governing bodies and lobby groups discuss in relation to the duty of care involving concussion management and return-to-play (RTP) protocols [6]. These data provide first-hand accounts that may inform current and future players who participate in rugby union and other contact sports.

### 2.6. Data Analysis

These data were reviewed and analysed thematically according to the Braun and Clarke’s [20,21,24] reflexive thematic analysis following an update to their original thematic analysis approach. The research team utilised an established critical realist framework to categorise and ascertain the ex-professional players’ descriptions of their experience of concussion management in professional rugby union.

### 2.7. Transcription

The original audio soundtrack of the interviews (via MS Teams) were transcribed verbatim by the lead researcher (E.D.). These transcripts were examined for exactness compared with the original audio recordings to cross-check and re-edit the transcripts. This procedure involved an initial familiarisation with the content of each transcript by re-reading each transcript on four separate occasions per interviewee. This practice enabled the lead researcher to confirm that the questions and responses interrelated with the audio soundtrack of the interviews. Another member of the research team reviewed all amendments and/or corrections to the final transcripts (L.R.).

### 2.8. Coding

The coding for this study focused on the semantic meaning of the interview transcripts. This was carried out by the first author (E.D.) and reviewed with another member of the research team (L.R.). The codes included the full range (both negative and positive) of concussion management experiences associated with being a professional rugby player. Theme development was guided by the first author (E.D.) in discussion with another author (L.R.). Initially, duty of care associated with concussion management, return-to-play protocols, and the use of protective equipment were identified as potential themes. Categories were first created to describe the raw data into meaningful units. Subcategories were then developed to denote the shared relationships between categories. The two overarching themes were then identified after further analysis of the sub-themes and their relationships (Table 1).

### 2.9. Researcher Background

The primary interviewer for the research was E.D. This collective decision made by the research team was based on his previous employment experience of being part of a professional rugby organisation in a coaching role. It was expected that the retired professional players would be more unreserved in their responses when compared to other members of the research team. This approach was supported, as it would be a male interviewer (E.D.) conducting the interviews with male participants. This was regarded as a positive characteristic with respect to the recruitment of participants for this study.

## 3. Results

Initial descriptive codes were categorised, and using further analysis, relationships between the codes were identified. This enabled the development of sub themes, which in turn lead to the classification of overarching themes. In this study, two major overarching themes were identified: (1) duty of care to professional rugby players by medical team, coaching staff, and the owners of professional clubs and (2) the use of protective equipment and law changes to enhance player safety of the field of play. These were based on the identified subcategories and the relationships between these and the subthemes (see Table 1).

## 4. Theme 1—The Duty of Care to Professional Rugby Players by Medical Personnel, Coaches, and Professional Clubs

### 4.1. Medical Interventions

The retired players had relatively consistent opinions regarding their personal experiences with medical teams within the professional clubs by whom they were employed. In many instances, the level of knowledge about concussion that the club doctor possessed was viewed as being central to the recognition and management of on-field concussion incidences. For example, *“…any medical staff that I worked with are always really on top of it (concussion) in that respect and there’s no dressing down the severity or the potential severity that concussion”* (P18) and *“…I feel like the medics, know the risks are so great with concussion, I suppose that they take a pretty hard line on it, and rightly so”* (P2).

The caveat to these opinions would be the level of experience that the medical teams possessed, whereby less-experienced personnel may be swayed by the players themselves or members of coaching staff. This point was illustrated by *“I guess it’s the fact that the matter is that with concussions are still quite an unknown entity and medical teams do their best”* (P12) or *“I do not remember the first 20 min of that game I remember being on the side-line and the doctor talking to me and I’m wondering why he was talking to me”* (P11). Difficulties arose were there were instances of more subtle concussion symptoms*: “…those were the probably the trickier ones to diagnose because you know while you have a physio there, and you know and, at the same time your physio, and that’s not their job (to diagnose concussion)”* (P11). Some players had a more severe perspective on concussion management during their playing careers: *“Hindsight is easy at this point, isn’t it? To look back and say how disgraceful it was (recognition and management of concussions), but it was just the way things were done”* (P5).

An overriding perspective that was discussed was the strength of character of the lead medical resource or the lead physiotherapist. Many players agreed that the medical team generally had the best interests of the players foremost in their actions. This protection of the players by the medical team not being influenced by coaching staff was admirable: *“I think for the time that I was under their care, I’ve never worked with a medical team that doesn’t care about their players”* (P12). With reference to medical staff that buffered coaching staff, *“I was lucky that medical staff I worked with were always strong enough personalities”* (P14) supported by *“So she (the doctor) had the respect (of the head coach/coaching staff) that she could make a comment on what was happening in the moment, and no one would argue”* (P16).

### 4.2. Concussion Testing and Return-to-Play (RTP) Protocols

It was the contention of most of the participants that concussion testing and RTP protocols were not robust enough to fully detect and manage concussions. While none of the participants were medically qualified, they felt able to manipulate the RTP system *“… (if you failed a test), you’d say nothing about it because you got another one (from the physiotherapist) the testing on the computers, that was a bit of a joke”* (P13). In addition, this point was expanded upon by another participant who stated *that “The concussion protocols you go in and you do the test with the playing cards and all that kind of stuff, I passed them (while symptomatic)”* (P22). The ability to manipulate test results may have had more serious consequences, as one participant noted that *“…it (concussion diagnostic procedure) was definitely not a robust process because I passed (while being concussed) it and then was able to just say jump in my car and drive home”* (P23).

A notable comment was that RTP protocols may have been influenced by the comments the players made to the medical team regarding their own recovery. These circumstances were exacerbated by players not fully disclosing the extent of their symptoms as they were progressing through the RTP protocol *“I remember going back jogging and in the early stages in the protocol and like even just that movement of a light jog was like I just felt woozy (and said nothing)”* (P6) and *“I remember the first training session back, I ended up getting sick (undisclosed) which you know is obviously a massive red flag”* (P6).

This occurred on multiple occasions particularly where the on-field doctor or physiotherapist was less experienced or less forceful in their stance to remove the *player “…. the (physiotherapist) came with a bottle and told me ‘Look, I think you need to come off’ and I’m telling the physio, no I’m fine”* (P8). The RTP protocols are widely accepted as most the established pathway for players to return to play after a suspected concussive incident. Many players expressed comments of concern regarding the accelerated RTP procedures that professional rugby players utilise. Even though recent changes (World Rugby 2022) to the duration of “stand down” time post concussive injury for a player has increased to twelve days, some players at the time of data collection felt that this may not be adequate:

“I can get knocked out on a Saturday and if he starts his RTP on a Sunday with no symptoms, then he can play again the following Saturday and I don’t think that that’s right. Like I mean, I think if a guy has visibly lost consciousness or has come close to losing consciousness, I don’t see how it’s acceptable that he’s back on the field seven days later if we’re being serious about it”. (P18)

As illustrated by the former players’ comments, the existing RTP protocols were not robust enough to ensure that their personal recovery was being managed thoroughly. Even though they were proceeding per the parameters of the RTP protocols, many players were not recovering within the specified RTP timeframe. Some players were enduring ongoing symptoms as they advanced through the protocols prior to returning to play:

“I remember about four, five days later (going through RTP protocols) I was leaving the facility and I walked out the front door and I just had no memory of where I parked the car. You know I’m just standing there for a second and I think that unsettled me”. (P11)

This was a common occurrence with the current RTP protocols at the time of gathering these data. Many players experienced “dark room syndrome” in an effort to recover quickly and become available for selection within the allocated RTP recovery period: *“I know a couple of them (fellow professional players) have literally been locked in dark rooms for two weeks”* (P14) and *“I’d go through periods (during RTP) particularly towards the end of the day as I was getting mentally fatigued where I need to go to the room and have a lie down”* (P12).

The concluding messages from the ex-players were that the RTP process needs to be “taken out of the players hands”. In this respect, they meant that the player should not have any influence over their recovery. The RTP process needs to be monitored by club medical teams and independent medics not associated with the rugby club. This may be facilitated by implementing a more impartial RTP system that is comprehensive, independent, and fully objective:

“There’s no (longitudinal) evidence to suggest that the current way in its current format actually gives clarity around whether a concussion is taking place or not. I would like to see further investigation into the merits of it because I think something like that takes the subjectivity out of it and makes everything as objective as possible”. (P17)

### 4.3. Coaching Staff and Head Coach

As professional rugby union clubs are a result-driven business, it must be understood that head coaches and coaching staff are contractually driven to deliver winning match results. In this respect, coaches aimed to achieve these outcomes by utilising the roster of players at their disposal to win matches. As has now been established, many players chose to play while physically injured or while being concussed. During the ex-player interviews, there was a broad spectrum of opinion about the influence or pressure applied by head coaches and coaching staff on players to compete while being either physically injured or remaining symptomatic with concussion. This is illustrated by the extremes of opinion: *“There’s never any pressure from coaches to stay on the pitch”* (P12) to *“There used to be a lot of pressure coming from the coaches to get back out onto the field”* (P16).

Many players explained that they experienced pressure to play while being symptomatic, as demonstrated by the following comments:

“But as you know there are situations where you have an overbearing coach. There may not be a strong personality in charge of medical and the coach gets what he wants, and you do hear stories of players getting forced back into playing, not forced back, but coaxed back into playing”. (P14)

This was further illustrated by head coaches or coaching staff coaxing players to continue to play: *“…you know you being used to people (coaches) telling you like there’s nothing wrong. It’s nothing to worry about, you know”* (P21). On occasion, the overbearing influence of coaches may not have aligned with what is best for the long-term health of the player. The main reason for this is that the coaches were under pressure to produce winning results.

“When I notice as a player you are paid by the club to play there’s pressures on the director of rugby too because of relegation playoffs, to make the top six, et cetera. Then your medical staff is basically subservient, they have to keep him happy”. (P 9)

Some players acknowledged that the coaching ethos they experienced evolved over time, whereby some felt that *“I feel when I was younger (player), there was a bit of negligence from coaches and the general conversation around concussion”* (P5). This “win at all costs” ethos of *“….they’re making decisions based on the business of winning games”* (P8) was replaced in many instances. The evolution of coaches moving from overbearing to accepting the opinions of the medical staff with regards the health status of the players was evident: *“I see a much greater awareness of the symptoms of concussion, the dangers, the concussion, the return to play protocol as they are far better adhered to…. it’s much safer”* (P6). This transition of coaching ethos may not always be done willingly, however:

“Sometimes you get the case of coach little bit old school, and he has, maybe have a few gripes about it, but ultimately, I think they know that it’s for the for the right reasons; you know that they want their best players available”. (P17)

As the ex-players expressed on numerous occasions, professional rugby union is a business, and as such, they were acutely aware that the head coach and coaching staff were ultimately reporting to club owners and shareholders. This created pressures on the coaches and coaching staff as their careers and contracts were dependent on attaining results in competitions: *“I think players need to feel comfortable that they’re not getting pressured to play, which is easier said than done because more often than not, it’s the coaches or the owners who put pressure on*” (P14).

### 4.4. Role of the Professional Rugby Club Owners

Intrinsic to the success of professional rugby clubs are the investors or outright owners of the clubs themselves. In many respects, the careers and contracts of the head coaches, coaching team, medical teams, and players are dependent on the patronage of the club owners. The ex-players interviewed for this current study were acutely aware of the authority of the club owners. This was expressed as a “hand in glove” approach, where the head coach or director of rugby was essentially the day-to-day representation of the club owners: *“Because of all the external pressures because of those subconscious agendas (implemented by the head coach) everybody has a boss”* (P21). The ex-players were employees of the club; however, some expressed concerns over the volume of games they had to play during a season:

“…there certainly has to be a little more regard for what actually serves the best interests of the players. Rather than just let lumping more games on top of them and squeezing the cash cow for a little bit more”. (P1)

This manifested as a clear understanding that owners had to sell tickets and provide entertainment for the club supporters to support their rugby club business: *“They’re dependent on a crowd coming in the gates and on entertainment…. that’s going to make it the best rugby experience on the weekend”* (P8). The was a pervading sense from the ex-players that the club owners dictated the club culture. Some of the ex-players had very positive experiences with some of the professional clubs based on how the club was operated by the club owners:

“So, it’s kind of building the trust and building a good culture (in the club). The best teams I played with you didn’t have any of that sort of unknown uncertainty (unstable contracts), it’s probably likely going to lead to better performance anyway”. (P16)

When players were traded after their contracts ended, it sometimes resulted in them being uncertain in terms of how a new employer (club) viewed player welfare. The ex-players were clear in relation to how they discussed a potential move to a new club:

“You go to different places where players are seen as assets in one place and they’re not in another club. You have owners that want to make the money at all costs, and you have owners that want to do it as best they can”. (P8)

This was further supported by comments such as 

“You know, if you had an offer from certain clubs and an offer from another club with a bad reputation (on managing player welfare), I think a lot of players that would choose to go to (a club with a better reputation)” (P12).

The predominant reason for joining a new club was influenced by perceptions of how well they (clubs) managed players whether they were injured or not. It was the broadly accepted opinion by the ex-players that the club owners have a duty of care to the players while they are representing the club and post retirement:

“They (club owners) should care for them when they’re there, and if something happens, care for them in the future. I certainly think it’s part of any organization, any sport, or any business, they should be able to look after their people and part of that would be warning about the current side effects or potential side effects (of repeated concussions or sub concussive impacts”. (P7)

Most players stated that club owners “have a duty of care to the players”, and these types of insights were reinforced by players stating the following:

“Absolutely they have responsibility. I think if you’re going to own a sports team, if you’re going to run a sports team, you’ve got to be responsible for your employees. It’s the same as any business or well-run business, it has empathy towards its employees and wants the best for them (in the long term)”. (P14)

The ex-players were adamant that the long-term health of themselves and their fellow retired professional goes beyond the playing contracts. It needs to be extended into the retirement phase of their lives, as many professional rugby players retire relatively early due to the highly attritional nature of professional rugby union. These views are relevant, as many ex-professional players retire with physical injury and with long-term mental health issues: *“It’s in your mental health, really. That is maybe where there’s a bit of a discrepancy...like I do think that first year or two when you finish, a player can really struggle”* (P20) or *“(I’d be) driving down a gravel road and remember a few times, trying to drive as slow as possible because the shaking of the car (blurring effects on vision)”* (P20). This point was supported by the necessity to provide resources for players that may be experiencing the effects of physical injury or mental health difficulties post retirement:

“There should be a certain amount of money or whatever resource is towards. I think this is probably something that might crop up in the next 10 years…...put money towards like a scheme which does help players who are really struggling”. (P14)

The consensus from the ex-players related to the duty of care from club owners post retirement was a level of relatively simple support. The suggestions were, for example, ongoing access to club physiotherapists or specialist doctors associated with the clubs. The principal reason for this was illustrated as follows:

“…. but like there is going to be a lot of broken men coming out of rugby, that’s just the facts I think, and they probably do need a lot more support than they have at the moment. I think there does always need to be a safety net for those players, especially when you leave the game and you’re used to a certain level of care”. (P15)

## 5. Theme 2—Use of Protective Equipment and Law Changes to Enhance Player Safety on the Field of Play

### 5.1. Attitudes towards Protective Equipment for Players

All the ex-players had worn various types of protective equipment throughout their playing careers. These included shin pads, shoulder padding, protective equipment for the groin, hand protectors, or padding around the hip area. By far the most used pieces of equipment were scrum caps (to protect their heads) and gumshields/mouthguards (to protect their teeth and mouth). Most players had worn either a scrum cap or a gumshield or a combination of both while playing professionally.

One player stated *that “…. to a certain extent, especially a well-made, well-constructed modern gumshield will really help lower the risk of getting certain concussions”* (P7). However, this was not a broadly accepted opinion by most of the ex-players. Other players declined the use of gumshields, as they found them difficult to use in terms of breathing. One player was wearing a gumshield and *“…had teeth knocked out with gum shield then as well”* (P5). The general opinion for gumshield use across the cohort of ex-players was that it was a personal choice. When a player had a large impact to the mouth or jaw area, the gumshield would not mitigate against concussion or concussion risk: “I can’t see I don’t think any head gear or gum shields are gonna help you” (P9).

The views on scrum caps were more clearly defined by the group of ex-players: *“Most guys are of the opinion that scrum caps themselves don’t actually mitigate concussion in any way, shape, or form”* (P18). Other players described them as “placebos” (P6, P15, P22) or when they were wearing a scrum cap, *“I did clash with (players) who were wearing scrum caps and they generally always came off worse”* (P1). Further inquiry clarified that most chose to wear some form of head protection in order to reduce abrasions to the skin or stop further cuts if they were protecting an existing injury: *“…so they’re really only good for cuts and abrasions; they don’t serve any purpose (in protecting against concussion)”* (P1), and *“it (scrum cap) was more after splitting my head open, I think and then having swollen ears”* (P6). The ex-players were sceptical in relation to scrum caps in mitigating the effects of concussive or sub concussive impacts. In this regard, the ex-players had well-established opinions*: “because they don’t stop your brain rattling around inside your head”* and *“I’ve heard a lot about the action of your brain inside your skull rather than impacts on the outside”* (P5) or *“get the impact going one direction and the impact sends him another direction and it’s actually not them hitting the ground. It’s the movement of their head at that speed”* (P6).

This broadly held position that scrum caps could not prevent a player’s brain from moving once impacts and collisions were experienced during a match provided a key insight. Conversely, some players believed that by wearing a scrum cap, it made them more cavalier during rugby matches: *“I’d be inclined to think if you’re wearing a scrum cap, you’re actually more inclined to be a little bit more aggressive, you might go into things a bit harder*” (P3). Some ex-players used the word “reckless” in relation to players wearing head protection. It led players to believe that they had a greater sense of safety: “…*and then you’ve got people saying that actually they encourage players to have an over sense of protection*” (P17) or “*I deliberately took it (scrum cap) off because he thought he was more cavalier and reckless with it on”* (P16). The overall sentiments on the use of scrum caps in professional rugby was exemplified in the following:

“…. You get this feeling of invincibility (wearing a scrum cap) and they (professional players) do tackle with their heads (and are) taught to tackle with their heads, which is just mind blowing in this day and age. It does in fact increase the extra risks they are taking, and it doesn’t stop anything in terms of the speed of the brain hitting the inside the skull”. (P14)

### 5.2. Improving on Field Safety for Players

As rugby is a contact sport, and there are specific playing positions on the field, such as outside centre or back row players, that are subject to high levels of physical contact throughout a game. This can naturally lead to some players being more exposed to injury risk than their teammates. Other aspects of the game carry an equally high risk of injury, for example, tackling, scrummaging, rucks, and mauls. Most players throughout the course of a game will be involved in these high-impact and potentially high-injury-risk situations. In recent times, World Rugby has attempted to address the level of injury risk exposure that players have during these contact situations by amending tackle height, amongst other measures [25].

As previously mentioned, many players believed that fewer matches throughout a season would reduce injury risk. This was supported by other players suggesting that less contact (e.g., tacking, scrummaging, rucks, etc.) during training sessions would further assist in reducing injury risk exposure: *“The game at the moment is basically just designed around being a bigger, stronger player… it’s just to be massive, powerful people running into each other… it doesn’t always have to be this massive collision thing”* (P15).

One aspect that World Rugby cannot influence is the ongoing ethos of developing larger players that will ultimately engage in larger collisions. This trend of developing a larger physical specimen of player reflects the current state of the game of rugby union. With more physically developed athletes competing, it translates into less space on the field of play and a higher injury risk. One player succinctly stated that *“rugby is coached around not conceding ground, so you’re kind of looking for big aggressive front on hits (tackles) all the time”* (P1).

When players are larger, one element of the game may be addressed to combat physical size with better contact skills in the collision areas of the game. This opinion was supported by ex-players who felt that an improvement in tackle technique would reduce concussion incidence in the tackle area: *“…I think you can improve around the tackle area by just coaching people, to be better on how to tackle”* (P21). There were other suggestions that may mitigate against concussion around the tackle area by limiting the number of players involved:

“…. if you can execute a far better tackle, in a more efficient (manner), it’s more likely to mean that you actually need less people to tackle. When you get that sort of multi-person tackle… it is gonna cause more concern when you got three or four players (in a tackle) … you are just increasing the chances of concussion”. (P16)

Recent research found that reducing the tackle height may reduce the frequency that rugby players receive an impact to their head or neck region. This research concluded that lowering tackle height resulted in an increase in concussions compared to standard tackle height [26]. In the participants for the current study, there were a variety of opinions related to the discussion of tackle height. Some ex-players stated that law changes may have little or no effect: “*He’s (the tackler) gonna get clipped by a hip or a knee or whatever and you lose your line of vision the lower you go*” (P5). Another shared such a view by stating the following:

“Lowering the tackle height further, which has been discussed, would be a mistake. I think that if you were to make everyone tackle below the waist, I think that’s going to lead to more concussions. Because the reality is for someone who is 6′6”, if you got to defend two players and all of a sudden, you make a last second decision (to make a tackle below the waist); my head is the thing that’s gonna take the bang (and get concussed)”. (P14)

The final consensus regarding tackling in the game was that there is no simple remedy to reduce the current rate of concussion in professional rugby union involving the person who instigates that tackle: “*it is very difficult to change (how to tackle) …. There aren’t many laws that you can change that are going to protect the tackler…. or the ball carrier*” (P14). This will continue to be the status quo of professional rugby union, as in many ex-players opinions, the reality is as follows:

“The game has become even tougher (levels of aggression) because people are adapting to the new laws, but they’re still getting bigger and stronger, you know, and the collisions are still getting bigger and stronger; the games are not getting softer”. (P17)

As mentioned, tackling is an area that generates most concussive incidents in the game [27]. However, there are other contact areas that involve a combination of tackling and general physical strength, such as scrummaging, mauling, and rucks. With many professional rugby union teams aiming to play a high-tempo style of play to retain possession, rucks are a high-risk injury area in the game [28]. Rucks and rucking involve high-speed collisions to “clean out” opposition players. This translates into highly athletic players accelerating into impacts using a large amount of physical strength and force to remove opposing players, which translates to an increase in injury risk [29].

One ex-player that had experienced multiple concussions throughout his career stated that *“… most of mine (concussions) came from cleaning out aggressively or getting cleaned out ‘cause you end up on your back*” (P21), which translates to players being in vulnerable positions and exposed to additional injury risk. This area of the game is difficult to monitor because of the number of variables that happen during rucks in the game of rugby union. World Rugby has attempted to change the laws of the game relating to rucks and the “breakdown”. Some ex-players believed these law changes did not mitigate against concussion or other injury risk, “*but whenever you step back and see the way the game is gone, since these laws were introduced, some people ask, ‘has it become safer?’ no, it hasn’t*” (P17). Alternatively, other ex-players suggested that strength coaches could mitigate against concussion risk by developing neck strength in their players: “*amount of posterior work around the neck within their program as a prevention strategy”* (P3) or “*I think exercise, probably neck strengthening and I don’t know enough about it, could possibly help reduce concussion*” (P9). These comments regarding neck strength were at odds with other statements about the mechanism of how the brain moves within the skull once a player is tackled or involved in other contact areas of the game.

## 6. Discussion

One of the primary objectives of this study was to assess the experiences of concussion management and RTP protocols for retired professional rugby union players. This study sought to understand the players’ perspective in relation to their personal experiences of concussion and how these were dealt with by the medical teams, head coaches (directors of rugby), support coaching staff, and professional ruby union clubs. As mentioned previously, all participants in this study had played professionally for several seasons, and all had experienced multiple concussions throughout their careers. In addition, the participants had witnessed many fellow professionals experience concussions during their careers and the long-term effects of concussion.

A consistent factor that emerged was that an experienced, well-respected medical lead was essential in any professional rugby union organisation. This was viewed as important for an obvious reason, i.e., a higher level of concussion knowledge and management experience may translate into a better level of care. A secondary reason was that a well-respected medical lead was a key component to act as a buffer between the players and coaching staff (including the head coach). It was evident during these interviews that players were coerced to return to play sooner rather than being given adequate time to recover post-concussion. This is propelled by the certainty that professional rugby union franchises are results-driven and financially focused businesses. Head coaches, coaching staff, and medical staff are employed by professional rugby organisations to deliver results to club owners and investors.

As has been reported previously in the literature, medical staff are generally seen as having the highest level of concussion knowledge and are encouraged to play the significant part in providing concussion awareness and education [30]. Even though this is generally accepted as the case, it remains challenging for medical staff to translate concussion knowledge for coaches, coaching staff, and players [31]. Due to a discrepancy in the understanding of the mechanism and severity of concussion, it has been reported that many players and coaches did not believe that concussion can impair performance [30]. As has been recorded in the literature, coaches can pressure medical staff to clear players to play and manipulate the RTP process [32,33]. Where medical staff and clinicians are reported to have pressured players to return prematurely, this type of practice will only further compromise the long-term health of the players in many facets. It is imperative that medical teams adhere to robust objective RTP protocols for the long-term health and wellbeing of the athletes under their supervision [34]. Players who have a history of concussion are twice as likely to experience other forms of physical injury compared to players who do not have a history of concussion [35].

As professional rugby clubs are established to be viable commercial businesses, it is paramount that they operate in an ethical manner [36]. Professional rugby clubs are unique due to the attritional nature of the sport. Simply expressed, this means that club owners and club administrators are required to properly manage the interests of their players first [37]. Previous research into this area has suggested that players view themselves as “commodities” and that injury was viewed as an “occupational hazard” within rugby union [38]. Many of the players interviewed for this current study believed that appropriate contract management, club culture, and having their long-term health needs considered are basic tenets for a well-managed professional club.

While they were competitive athletes, the most effective tool to protect the players from undue exposure to injury risk was by managing workload. From this perspective, all stakeholders involved in running professional rugby clubs need to manage the physical demands that players are exposed to over the course of their careers [11]. This spectrum of player responsibility includes head coaches, coaching staff, medical teams, and professional club owners.

Emerging evidence has found that many ex-professional rugby union players are experiencing long-term physical injury and long-term cognitive damage as a result of playing the game professionally [17,39]. All participants in this current study had multiple concussions (diagnosed and undiagnosed) throughout their professional playing careers. Research by Rafferty et al. [40], which focused on professional rugby union, highlighted that players were more likely to be concussed than not concussed after 25 competitive matches (7.9 (95% CI 5.1 to 11.7) to 21.5 injuries/1000 player-match-hours (95% CI 16.4 to 27.6) over the duration of four playing seasons). This research also found that after suffering a concussion, players had a 38% greater injury risk for another concussion compared with a non-concussive injury [40]. Comprehensive responsibility by club owners, head coaches, coaching teams, and medical teams must be viewed as essential to managing player workload for long-term health even in retirement. Furthermore, part of a player’s consideration during contract negotiations is how they perceive player welfare issues are being managed within a club. Exhibiting a high level of player welfare and long-term duty of care policies could have an impact on a club’s ability to recruit prospective players.

Most players interviewed had used protective equipment during their playing days. The overriding opinion by the participants was that these types of products did not mitigate against concussive impacts either while training or while playing competitive matches. There is strong evidence to support the players’ opinions. For example, research by [26] found that scrum caps did not reduce the odds of concussion in professional rugby union (adjusted odds ratio = 1.05, 95% CI: 0.71–1.56). There are many misconceptions about the protective merits of wearing scrum caps. For example, in contrast to the intended purpose of encouraging players to play in a safer manner, scrum caps can induce players to play more aggressively [41]. These points were mentioned by the participants in this study, as they used words such as “reckless” and “cavalier” when discussing scrum caps. This suggests that greater evidence needs to be provided via longitudinal studies regarding the effectiveness of scrum caps on concussion mitigation. Currently, study of many scum caps claims to reduce linear or rotational forces to the scum cap itself; however, these research papers do not prove that this prevents the brain from moving within the cranium of the player [42].

Since 2019, World Rugby has made attempts to alter player behaviour in relation to tackle height and, more specifically, high tackles. With the introduction of the high tackle sanction framework (HTSF), there was a notable increase in high tackle sanctions (58%), but a visible improvement in player behaviour regarding height tackles was not observed [43]. Strict adherence by referees to the laws of tackle height and adaptation by head coaches and coaching staff are required to reinforce this necessary change in player behaviour [43].

Other experts in the area of tackle technique have proposed that either reducing the speed of entry to contact situations such as tackling, and rucks may reduce the odds of injury risk [27]. Tackles to the torso and chest areas of players has been viewed as a successful way to defend against offloading by the ball carrier. This practice can lead to an increase in head-on-head contact between defenders and attackers [44]. When players are coached to tackle lower, there will be an increase in offloads, but this may translate to safer tackle techniques for the defender and the ball carrier, and this lower tackling may assist in changing player behaviour with respect to tackling [44].

## 7. Conclusions and Limitations

Professional rugby union is a highly physically demanding field sport. It is imperative that those who are responsible for the long-term welfare of the athletes who play the game do so in a holistic manner that protects the players. Many of the players who were interviewed for this study stated that all decisions about RTP should be taken out of the players’ hands and emphasized the need for the implementation of a more stringent concussion testing protocol. In addition to this, many ex-players would prefer a longer RTP process. Professional players will not disclose injury for a variety of reasons, as they are highly competitive individuals who want to retain or obtain lucrative playing contracts. In turn, the long-term health of players must be monitored by medical staff, coaching staff, head coaches, and club administrators. There is mounting evidence to suggest that there are long-term effects of repetitive head impacts, leading to detrimental effects in the cognitive abilities and neurological health of current and past players.

It is reasonable to say that players need to be protected in terms of workload management and from themselves. A fuller understanding of head kinematics and/or tackle height would be beneficial along with an adjustment of player behaviour in the contact areas of the game. These may assist in reducing the incidence of concussion or injury risk in rugby union. Other approaches to making the game safer may be derived from different coaching practices centred on the ball carrier’s responsibility in the tackle situation. There are limitations to the current study, as the cohort had retired in the past ten years. During this time, there have been changes and recommendations to the laws of the game and to the level of workload experienced by existing rugby union professionals. These changes may have an impact on long-term injury issues for future professional players.

Finally, as described by the players themselves, further work by professional clubs is required to ensure the highest duty of care is delivered not only to currently listed players but also recently retired players to safeguard those who have contributed so much to the sport’s growth and success. Future research will seek to explore the current culture of concussion management in professional rugby.

## Figures and Tables

**Table 1:** Results of thematic analysis of retired professional male rugby player perceptions of duty of care towards players and protective measures in the game (*n* = 23).

Themes	Categories	Subcategories	Sample Quote
1. Duty of care to players to players by medical personnel, coaches, and professional clubs	Medical interventions	Influence of medics on concussion diagnosis and intervention	“…. they took me off the pitch so you know you’re relying on really, really good medical help to step in when you’re not fully with it.” (P4)
		Strong personalities in the medical team, knowledgeable, under pressure to manage, lack of experience	“Any medical staff that I worked with and are always really on top of it in that respect and there’s no dressing down the severity or the potential severity that concussions can have.” (P18)
		Lack of resources or experience	“They were very stretched so that was probably a pitfall. I think if I potentially have been looked after a little bit better, my career would have went on longer.” (P5)
	Concussion testing and return-to-play (RTP) protocols	Improving testing, not robust enough, subjective testing	“The concussion protocols you go in and you do the test with the playing cards and all that kind of stuff, I passed them (while symptomatic).” (P22)
		Setting lower baseline tests, intentionally scoring lower	“I remember actively practicing those tests, trying to figure out the months of the year backwards learning that so I could do it. I could go into autopilot and beat those tests.......you can beat the system.” (P15)
	Coaching staff and head coach	Influence of coaches on medical staff decisions and player readiness	“No definitely not, and not every player was as honest, some players were more honest and not every coach was happy for the medical staff to make the decision.” (P8)
		Players coaxed to play or no pressure from coaches	“There are situations where you have an overbearing coach and maybe not as strong of a personality in charge of medical and the player does not get forced back, but coaxed back into playing.” (P14)
	Role of the professional rugby club owners	Sustainable business operations	“If you’re going to run a sports team, you’ve got to be responsible for your employees. It’s the same as any business you any well-run business, uh, as empathy towards its employees and wants the best for them.” (P14)
		Duty of care and long-term duty of care	“But like there is going to be a lot of broken men coming out of rugby, that’s just the facts I think, and they probably do need a lot more support than they have at the moment.” (P15)
2. Use of protective equipment and law changes to enhance player safety on the field of play	Attitudes towards protective equipment for players	Use of protective equipment (scrum caps and gumshields)	“I don’t think scrum caps themselves can have much of an effect, I think it may help with pain of head collisions and obviously with cuts and everything else and to avoid getting split.” (P18)
		Reckless behaviour	“Deliberately took it off because he thought he was more cavalier and reckless with it on (scrum cap).” (P16)
	Improving on field safety for players	Tackle technique	“We can coach our young athletes in in their tackle technique as well, I think will be a would be a big defining factor.” (P2)
		Strength training and neck strength	“I think exercise, probably neck strengthening…but I don’t think any head gear or gum shields are going to help you.” (P9)
		Law changes in contact areas	“Players flying into rucks and taking lads’ heads off, that’s probably something that could be looked at ‘cause it is quite dangerous.” (P5)
		Reducing tackle height	“There aren’t many rules that you can change that are going to protect the tackler.” (P14)
